# A case of SFTS coinfected with *E. coli* bacteremia

**DOI:** 10.1186/s12879-020-05705-0

**Published:** 2021-01-07

**Authors:** Hyungdon Lee, Woo Young Choi, Choon Mee Kim, Na-Ra Yun, Dong-Min Kim, Sang-Hyun Pyun, Byung Jun Yu, You Mi Lee

**Affiliations:** 1 Department of Internal Medicine, Chuncheon Sacred heart hospital, College of Medicine, Hallym University, Chuncheon, South Korea; 2 Department of Plastic and Reconstructive Surgery, College of Medicine, Chosun University, Gwangju, South Korea; 3 Premedical Science, College of Medicine, Chosun University, Gwangju, South Korea; 4 Department of Internal Medicine, College of Medicine, Chosun University, 588 Seosuk-dong, Dong-gu, Gwangju 501-717 Republic of Korea; 5 Graduate School of Chosun University, Gwangju, Republic of Korea

**Keywords:** Severe fever and thrombocytopenia syndrome phlebovirus, *Escherichia coli*, Coinfection

## Abstract

**Background:**

Severe fever thrombocytopenia syndrome virus (SFTSV) is the causative agent of severe fever thrombocytopenia syndrome (SFTS). SFTS is an emerging infectious disease, characterized by high fever, gastrointestinal symptoms, leukopenia, thrombocytopenia, and a high mortality rate. Until now, little importance has been given to the association of SFTS with leukocytosis and bacterial co-infection.

**Case presentation:**

A 51-year old man visited our hospital with fever and low blood pressure. He was a farmer by occupation and often worked outdoors. He had a Foley catheter inserted due to severe BPH. Laboratory tests revealed thrombocytopenia, elevated liver function, and elevated CRP levels. He had marked leukocytosis, proteinuria, hematuria, and conjunctival hemorrhage. Initially, we thought that the patient was suffering from hemorrhagic fever with renal syndrome (HFRS). However, we confirmed SFTS through PCR and increasing antibody titer. However, his blood culture also indicated *E. coli* infection.

**Conclusion:**

SFTS displays characteristics of fever, thrombocytopenia, elevated liver function, and leukocytopenia. We described a case of SFTS with leukocytosis due to coinfection with *E. coli*. Since patients with SFTS usually have leukocytopenia, SFTS patients with leukocytosis are necessarily evaluated for other causes of leukocytosis. Here, we report the first case of an SFTS with concurrent *E. coli* bacteremia.

## Background

Severe fever with thrombocytopenia syndrome (SFTS) is an emerging viral disease with a high mortality [1]. This disease was first reported in China in 2011 [1]. In China, SFTS generally occurs from May to August, but in South Korea, SFTS patients are increasingly presenting between September and October because of the traditions of weeding graves during the thanksgiving period and increased outdoor activity during the harvest season [2, 3].

The incubation period of the SFTS is 4–15 days followed by major clinical manifestations of high grade fever, myalgia, gastrointestinal symptoms, thrombocytopenia, leukopenia, lymph node enlargement, raised hepatic enzymes, neurological disorders and occasionally coagulopathies and the estimated mortality rates of SFTS cases are proportionately high in South Korea (21.8%) followed by Japan (18.8%) and China (4.8%) [1, 2, 4]. In South Korea, the yearly incidence of SFTS cases gradually increased from 36 cases in 2013 to around 212 cases in 2017 [5]. In the years 2013 to 2016, the SFTS prevalence rate in this region was 1.19 per 100,000 inhabitants [6].

Studies showed that, the majority of patients (86–97.5%) with SFTS are accompanied by leukopenia [1, 7]. However, until now, little is known about association of SFTS with leukocytosis and bacterial co-infection. In this study, we report a case of SFTS with leukocytosis due to *Escherichia coli* bacteremia.

## Case presentation

**Fig. 1:**
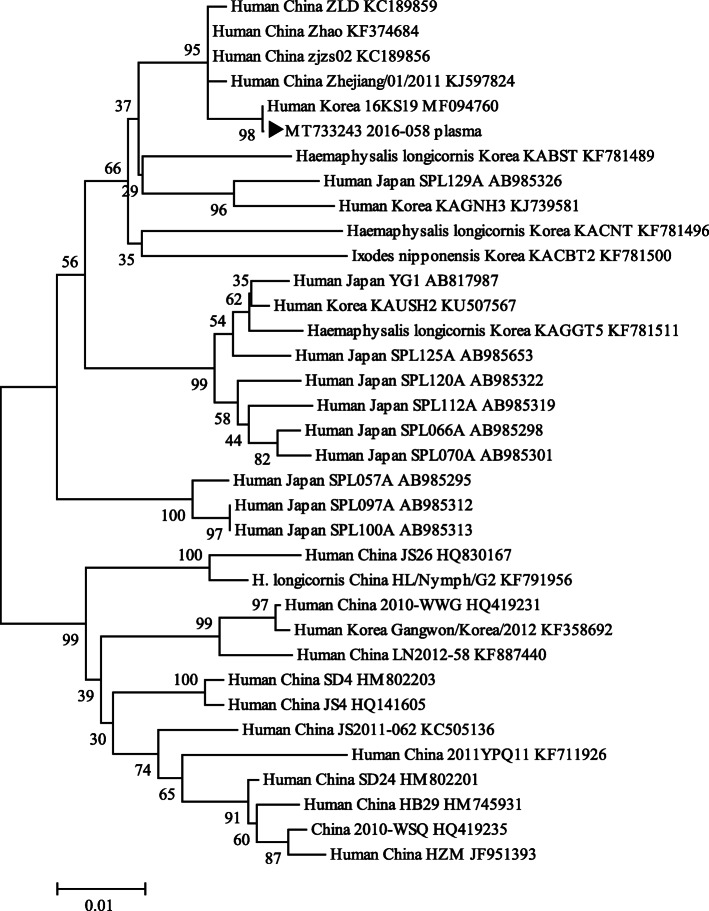
Phylogenetic tree based on the SFTSV M segment partial sequences from SFTS-positive patient specimens. Phylogenetic tree based on the SFTSV M segment partial sequences (477 bp) from GenBank and SFTS-positive patient specimens (▸). Scale bars indicate 0.01 base substitutions per site. GenBank accession numbers are shown in the tree

A 51-year old male was admitted to Chosun University Hospital with fever and low blood pressure. He was treated with a hepatitis B and C antiviral agent, and had a Foley catheter placed due to severe benign prostatic hyperplasia (BPH) for 2 months before admission. When he arrived at the hospital, his vital signs revealed hypotension (40/20 mmHg) and fever (37.9 °C). Laboratory tests revealed thrombocytepenia (100,000/μL) and elevated liver function [Aspartate aminotransferase (AST) 131 U/L, Alanine aminotransferase (ALT) 95.9 U/L], elevated C-reactive protein (CRP) (6.03 mg/dL), decreased renal function [Blood urea nitrogen (BUN) 28.5 mg/dL, Creatinine 2.54 mg/dL], hematuria 4+ (RBC many/high-power field [HPF]), proteinuria 2+, and WBC 5–9/HPF in urine analysis. He was a farmer by occupation and therefore worked outdoors. He had marked leukocytosis (24.83 × 10^3^ /μL), elevated procalcitonin (100 ng/mL), hematuria, and conjunctival hemorrhage. Therefore, we thought that the patient had an acute febrile illness during the autumn season, such as hemorrhagic fever with renal syndrome (HFRS). PCR tests and antibody tests for *O. tsutsugamushi* and Hantavirus virus were performed, but all were negative. We initiated treatment for septic shock, followed by Hanta virus, *L. interogans,* and *O. tsutsugamushi* serologic follow-up tests. However, all results were negative. Nested reverse-transcription polymerase chain reaction (nested RT-PCR), real-time RT-PCR, and indirect immunofluorescence assay (IFA) were performed based on the suspicion of SFTS (Tables 1 and 2) [8, 9]. The nested RT-PCR targeting M segment was positive and real-time RT-PCR targeting the S segment (Ct value of 41.21) was negative. DNA sequence analysis revealed the presence of SFTS virus in the patient specimens. Sequence similarity analysis with the M segment partial sequences of the PCR product (2016–058 plasma) showed 99.2% similarity (473/477) with the SFTS virus strain 16KS19 isolated from a Korean patient (accession no. MF094760.1). The phylogenetic tree also showed that the M segment partial sequence from the specimen formed a cluster with SFTS virus strains (Fig. 1).

**Table 1:** Nested RT-PCR and real time RT-PCR primers and probe used in this study

Target gene	PCR	Primer name	Sequences	Product size (bp)
M segment	Nested RT-PCR (1st PCR)	SFTS-M 1st-F	TCATCCTGACYTATTYTGCAATWG	640
		SFTS-M 1st-R	TAAGTYACACTCACACCCTTGAA	
	Nested RT-PCR (2nd PCR)	SFTS-F (=MF3)	GATGAGATGGTCCATGCTGATTCTAA	560
		SFTS-R (=MR2)	CTCATGGGGTGGAATGTCCTCAC	
S segment	Real-time RT-PCR	SFTS-SQ-F	ACCTCTTTGACCCTGAGTTWGACA	120
		SFTS-SQ-R	CTGAAGGAGACAGGTGGAGATGA	
		SFTS-SQ-P	[FAM]-TGCCTTGACGATCTTA [NFQ-MGB]

**Table 2:** SFTS diagnostic test results with IFA, nested RT-PCR, and real-time RT-PCR

Date	IFA		M segment targeting nested RT-PCR	S segment targeting real time RT-PCR
	IgG	IgM		
7–1	IgG < 32	IgM < 32	positive	41.21
7–8	IgG < 32	IgM < 32	negative	Undetermined
7–11	IgG < 32	IgM < 32	negative	
7–21	IgG 32	IgM < 32		
8–16	IgG 64	IgM < 32

The SFTS IFA test results revealed immunoglobulin M (IgM) and IgG titers that were both < 1:32 at the time of admission. However, in follow-up IFA tests, the IFA IgG antibody titer was 1:64. However, he continued to have leukocytosis (Table 3). The blood culture was performed on the day of admission, following six days, results showed the *E. coli*. To evaluate the portal of entry of *E. coli,* the abdomen and pelvis computed tomography and urine culture was performed, the result showed no sign of infected focus or bacterial growth respectively. However, the result of urine culture was reported 4 days after admission, after which we discarded the urine specimen and no further cultivation was performed. Therefore, the urinary tract as a potential entry portal for *E.coli* cannot be clearly ruled out.

**Table 3:** Complete blood cell count

Date	White blood cell (μL)	Hemoglobin(g/dL)	Platelet count (μL)
Admission day	25,240	12.6	81,000
Day 3	36,670	10.7	50,000
Day 4	27,540	10.6	62,000
Day 5	7520	12.1	65,000
Day 7	10,880	12.0	128,000
Day 12	6580	9.7	498,000
Day 14	6080	10.4	621,000

We treated the patient for septic shock due to *E. coli* bacteremia, including ceftriaxone and inotropic. On the third day of hospitalization, his vital signs stabilized. Follow-up blood culture was performed on the fifth day of hospitalization, which revealed no growth of bacteria.

### PCR amplification

At the time of presentation, viral RNA was extracted from the patient’s plasma using a Viral Gene-spin Viral DNA/RNA Extraction Kit (iNTRON Biotechnology, Sungnam-si, Korea). For the nested RT-PCR, cDNA was synthesized using SuperScript VILO MasterMix (Invitrogen, CA, USA). The nested RT-PCR targeting the M segment of the SFTS virus was conducted using synthesized cDNA, M segment-specific primers, AmpliTaq Gold 360 Master Mix (Applied Biosystems, Foster City, CA, USA), and a Biosystems Veriti 96-Well Thermal Cycler (Applied Biosystems, Foster City, CA, USA) [8].

For the real-time RT-PCR targeting the S segment of the SFTS virus, previously reported primers and probes targeting the S segment were used [9]. The PCR primers and probes used in the present study are described in Table 1.

### Nucleotide sequencing and phylogenetic analysis

The positive PCR product was purified and sequenced in both directions, using the PCR primers and an automatic sequencer (ABI Prism 3730XL DNA analyzer, Applied Biosystems) at COSMO GENTECH (Deajeon, Korea). The sequencing results were analyzed using the BlastN (Bethesda MD, USA) network service from the National Center for Biotechnology Information (National Institutes of Health). Moreover, the M segment sequences obtained from the GenBank database and patient samples were used to construct a phylogenetic tree using ClustalX (Ver 2.0; www.clustal.org/) and Tree Explorer programs (DNASTAR, Madison, WI, USA). Bootstrap analysis was conducted using 1000 replicates to improve the confidence level of the phylogenetic tree.

### Indirect immunofluorescence antibody assay

To perform the IFA, Vero E6 cells infected with SFTS virus were inoculated and fixed with 80% acetone on Teflon-coated well slides to prepare the SFTS antigen slide. The patient serum was serially diluted two-fold and then reacted with viral antigens in a moist chamber for 30 min at 37 °C. After washing with phosphate-buffered saline (PBS, pH 7.2) and distilled water, fluorescein isothiocyanate (FITC)-conjugated anti-human IgG and IgM as secondary antibodies (MP Biomedicals, Ohio, USA) were applied to each slide and incubated at 37 °C in a humid chamber for 30 min. Then, the slides were observed using a fluorescence microscope (Olympus IX73, magnification: 400×) after dispensing the mounting solution (VECTOR Laboratories). The final serum dilution, which indicates the specific fluorescence, was used to determine the antibody titer [10].

## Discussion and conclusion

Leukocytopenia and thrombocytopenia in SFTS are typical laboratory findings consistent with decreased lymphocyte cellularity of the red pulp in the spleen and increased megakaryocytes in the bone marrow during the early stage of SFTSV infection [11]. Viral RNA of SFTS is in the spleen, and pathologic changes of spleen developed in the early stage of infection suggested that the spleen was the main target of the SFTSV. SFTSV co-localized with platelets in the cytoplasm of macrophages in the spleen, adhered to mouse platelets, and facilitated phagocytosis of platelets by macrophages in a mouse model, which suggests that SFTSV-induced thrombocytopenia is caused by clearance of circulating virus-bound platelets by splenic macrophages [11]. It is hypothesized that vascular endothelial injury might also contribute to thrombocytopenia and leukopenia, two important features of SFTSV infection. In addition, activated endothelial cells interact with white blood cells (WBCs) by the adhesion of WBCs to activated endothelial cells and their transmigration into interstitial space [12]. These reasons may explain why leukopenia is common in SFTS.

However, in this case, leukocytosis in the peripheral blood over 24.8 × 10^3^ /L was observed. This finding was very unusual for patients with SFTS.

One of the reasons for leukocytosis in this patient was *E. coli* bacteremia. Various factors that stimulate the bone marrow, such as granulocyte colony-stimulatory factor, adhesion molecules, and various cytokines (e.g., interleukin-1, interleukin-3, interleukin-7, and tumor necrosis factor) factor into the development of leukocytosis in the peripheral blood [13]. In this patient, SFTS coinfected with *E. coli*. Therefore, inflammatory cytokines and stimulatory factors could affect the bone marrow, and finally, peripheral blood leukocytosis was observed.

The second reason for leukocytosis in this patient was the effect of coinfection by bacterial and viral pathogens such as *E. coli* and SFTSV. In a case of coinfection with *Salmonella enterica* and norovirus, Salmonella infection reduced viral replication by blocking virus entry early in the virus life cycle and inducing antiviral cytokines later in the infection [14]. Similar to the coinfections by bacteria and virus pathogens mentioned above, we speculate that *E. coli* infection suppresses SFTSV replication. Then, it presented leukocytosis in the peripheral blood. However, this hypothesis requires further study.

In summary, leukopenia and thrombocytopenia are common in SFTS. However, when leukocytosis is revealed in patients with SFTS or epidemiologically suspected SFTS, it is necessary to evaluate co-infections with HFRS or bacterial diseases including urinary tract infection, pneumonia, and deep seated infection.

This case is the first case in Korea of an SFTS coinfection with *E. coli*.

## Data Availability

The datasets used and/or analysed during the current study are available from the corresponding author on reasonable request.

## Abbreviations

SFTS: Severe fever with thrombocytopenia syndrome
WBCs: White blood cells,
PCR: Polymerase chain reaction
nested RT-PCR: Nested reverse-transcription polymerase chain reaction
IFA: Indirect immunofluorescent assay
FITC: Fluorescein isothiocyanate
CRP: C-reactive protein
BPH: Benign prostatic hyperplasia.
